# Synthesis of Sn/Ag–Sn nanoparticles *via* room temperature galvanic reaction and diffusion†

**DOI:** 10.1039/c9ra02987g

**Published:** 2019-07-12

**Authors:** Min Jia Saw, Mai Thanh Nguyen, Shilei Zhu, Yongming Wang, Tetsu Yonezawa

**Affiliations:** Division of Materials Science and Engineering, Faculty of Engineering, Hokkaido University Kita 13 Nishi 8, Kita-ku Sapporo 060-8628 Japan tetsu@eng.hokudai.ac.jp

## Abstract

Tin (Sn) has a low melting temperature, *i.e.*, 231.9 °C for the bulk, and the capability to form compounds with many metals. The galvanic reaction between Sn nanoparticles (NPs) as the core and silver nitrate at room temperature under argon gas in an organic solvent without any reducing power, was employed for the first time to coat an Ag–Sn intermetallic shell, *i.e.*, Ag_3_Sn and/or Ag_4_Sn, on Sn NPs. For spherical Sn NPs, the NPs retained a spherical shape after coating. Uniform and Janus structures consisting of a β-Sn core with Ag–Sn shell were observed in the resulting NPs and their population related to the input molar ratios of the metal precursors. The observation of the intermetallic shell is general for both spherical and rod-shape Sn NPs. The formation of the intermetallic shell indicated that two reactions occurred sequentially, first reduction of Ag ions to Ag atoms by the Sn core, followed by interdiffusion of Ag and Sn to form the Ag–Sn intermetallic shell.

## Abstract 2

Coating of Ag–Sn intermetallic compound on Sn nanoparticles at room temperature.

## Graphical abstract

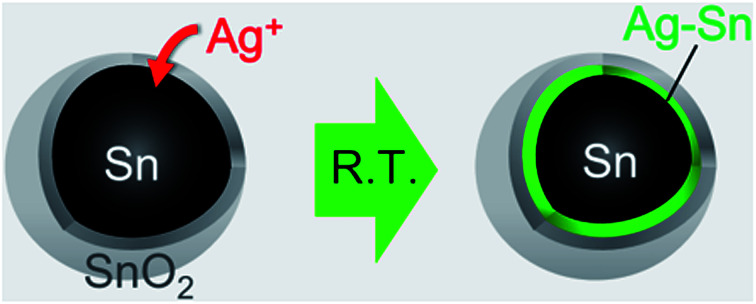

## Introduction

Sn nanoparticles (NPs) have been used as phase change materials,^1^ for gas-sensing,^2^ conductive inks for printed electronics,^3,4^ lead-free solder in electronics and anode material in lithium and sodium-ion batteries.^5–8^ Similar to various alloy NPs,^9–12^ the catalytic properties,^13–17^ conductivity,^18,19^ and soldering properties^20,21^ can be further modified and improved by introducing Ag into the Sn NPs to form Ag–Sn alloy NPs.^13–16,18–21^ The catalytic properties of Ag–Sn NPs were superior compared with that of Sn.^13,15^ In the work by Jo *et al.*,^19^ highly conductive Ag–Sn bimetallic NPs with various compositions (*e.g.*, 80Ag20Sn, 60Ag40Sn, 40Ag60Sn and 20Ag80Sn) were synthesized using the polyol method. The synthesized NPs had low sheet resistance which could be applied in printed electronics.^19^ Jiang and coworkers have observed that the Ag–Sn alloy NPs had lower melting temperatures with decreasing particle size.^20,21^ The low melting point NPs were suitable as solders in lead-free interconnects.^20,21^

Ag–Sn intermetallic alloy NPs have been synthesized *via* direct co-reduction of Ag and Sn at elevated temperature.^19^ In contrast, the reduction of Ag *via* galvanic reaction with Sn NPs and formation of Ag–Sn intermetallic NPs have been observed at 90 °C in oleylamine and 45 °C in hexadecylamine *via in situ* X-ray diffraction during the galvanic exchange reaction and elemental mapping of the product.^22^ The results show the growth of Ag–Sn part and a higher atomic concentration of Ag compared to Sn in the NPs after the exchange reaction, revealing the transformation of Sn to Ag–Sn intermetallic compounds (ε-Ag_3_Sn or/and ζ-Ag_4_Sn).^22^ The formation of Ag–Sn intermetallic compound can also occur at room temperature when depositing 100 nm-thick Sn on 50 nm-thick Ag film *via* a vacuum evaporation method.^23,24^ However, to-date, a selective coating of Ag–Sn intermetallic compounds on Sn NPs at room temperature has not been reported. The coating of Sn with less active metals at high temperature is challenging as it results in a structure consisting of the less active metal as the core and Sn, which is more active as the shell. We have observed that high temperature coating of Sn NPs with Ag resulted in normal core/shell structure with intermetallic Ag–Sn as the core, similar results with Kreigner *et al.*'s study,^22^ and Sn as the shell (Fig. S1†).

Thus, in the present study, β-Sn NPs were coated with Ag–Sn intermetallic compounds *via* a galvanic reaction of Sn NPs and Ag cations at room temperature. The resulting structure is a reverse core/shell, where Sn is the core and Ag–Sn is the shell. The surface coating of Sn NPs was carried out in *tert*-butyl alcohol (TBA), which does not have any reducing power. With TBA, the reduction of Ag cations by Sn NPs was solely performed *via* the galvanic reaction due to the difference in potential between Sn and Ag. Since no additional reducing agent was used to reduce Ag^+^ ions, the absence of Ag NPs as a byproduct after synthesis was expected. Room-temperature reactions and diffusion are beneficial for obtaining the reverse core/shell structure. The produced NPs were studied in detail to understand the reaction between Ag^+^ ions and Sn NPs at room temperature.

## Experimental

### 2.1. Chemicals for spherical Sn NPs and Sn/Ag–Sn NPs

Oleylamine (Kanto), tin(ii) chloride anhydrous (Junsei), tin(ii) acetate (Wako), lithium bis(trimethylsilyl)amide (Sigma Aldrich), diisobutylaluminium hydride (DIBAH) in 1.0 M toluene (Kanto), poly(vinylpyrrolidone) (PVP) K-90 with MW 630 000 (TCI), sodium borohydride (Wako), *tert*-butyl alcohol (TBA, Wako), silver nitrate (Junsei), and 1-propanol (Junsei) were used as received.

### 2.2. Synthesis of Sn NPs as the core

Spherical Sn NPs as the core were synthesized based on the modified method of Kravchyk *et al.*^5^ Sn nanorods (NRs) were also synthesized as the cores by using our previously reported method.^25^ Details of the synthesis procedures are given in the ESI.†

### 2.3. Preparation of Sn/Ag–Sn NPs

First Sn NPs of 0.25 mmol Sn atoms were dispersed in 20 cm^3^ TBA and sonicated for 10 min to form a dispersion of Sn NPs (0.0125 M Sn atoms). At the same time, a 0.0050 M silver nitrate stock solution was prepared in a vial by adding 8.5 mg silver nitrate to 10 cm^3^ TBA. The sonicated Sn NP dispersion was then purged under argon gas for 15 min, followed by injection of 1 cm^3^ silver nitrate stock solution. The solution was stirred at 500 rpm and reacted for 15 min at room temperature, *i.e.* 26 °C. The solution was then centrifuged using 1-propanol at 8000 rpm for 10 min. This purification step was repeated twice, and the purified Sn/Ag–Sn NPs were dispersed in 1-propanol for further characterization. Coatings of Sn NPs using 1 cm^3^ of 0.0125 M and 0.0230 M silver nitrate stock solutions were also performed as summarized in Table 1.

**Table 1:** Concentration and amount of Sn and Ag used in coating experiments

Sn NPs dispersion		AgNO_3_ solution		Mole ratio of Sn : Ag^+^
Sn at. concentration (M)	Sn at. amount (mmol)	Concentration (M)	Amount (mmol)	
0.0125	0.250	0.0050	0.0050	1 : 0.020
0.0125	0.250	0.0125	0.0125	1 : 0.050
0.0125	0.250	0.0230	0.0230	1 : 0.092
Sn NRs dispersion		AgNO_3_ solution		
0.0050	0.385	0.0892	0.4460	1 : 1.158

For the purpose of coating Sn NRs, 0.385 mmol Sn was dispersed in 77 cm^3^ TBA and then sonicated for 5 min to form a dispersion of Sn NRs with Sn atomic concentration of 0.0050 M Sn. At the same time, 0.0892 M silver nitrate stock solution was prepared in a vial by adding 0.3788 g silver nitrate into 25 cm^3^ of TBA. The sonicated Sn dispersion was purged under argon gas for 15 min while stirring at 800 rpm. 5 cm^3^ silver nitrate stock solution was then injected into the solution and left for 3 h at room temperature to allow the reaction to proceed. After reaction, the solution was centrifuged using 1-propanol at 15 000 rpm for 30 min. This purification step was repeated three times, and the product was dispersed in 1-propanol for further characterization.

### 2.4. Characterization

The crystalline and phase structures of the NPs were characterized using X-ray diffraction (XRD, Rigaku Miniflex II X-ray diffractometer, Cu Kα radiation, *λ* = 1.5418 Å, scanning speed of 2° min^−1^). XRD samples were prepared by dropping ∼50 mm^3^ dispersion of NPs onto the XRD sample plate. The morphologies and selected area electron diffraction (SAED) of the NPs were examined by a transmission electron microscopy (TEM, JEOL JEM-2000FX, 200 kV). High-resolution (HR) TEM and HAADF images were acquired using scanning transmission electron microscopies (STEM, FEI Titan Cube, 300 kV and ARM200F, 200 kV). The TEM sample was prepared by releasing a drop of NPs dispersion onto a collodion-coated copper TEM grid. TEM/STEM-energy dispersive X-ray spectroscopy (EDX) was used to analyse the composition and elemental distribution of the NPs. The average size of NPs was estimated from 150 NPs in an arbitrarily chosen area of enlarged TEM images.

## Results and discussion

### 3.1. Crystal structure and morphology of Sn/Ag–Sn NPs

**Fig. 1:**
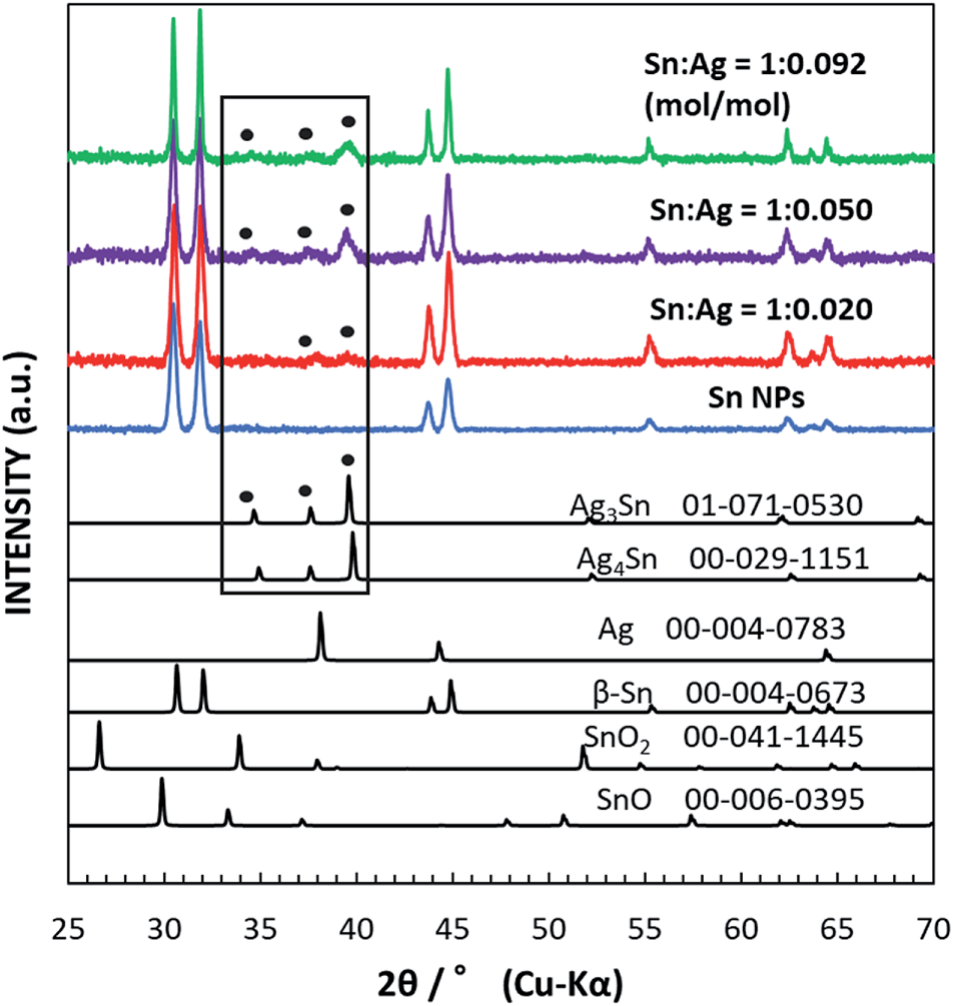
XRD patterns of the as-synthesized Sn NPs (blue curve) and Sn/Ag–Sn NPs with various input molar ratios of Sn and Ag, *i.e.*, Sn : Ag = 1 : 0.020 (red curve), 1 : 0.050 (violet curve), and 1 : 0.092 (mol/mol) (green curve). Reference patterns of β-Sn (JCPDS no. 04-0673), SnO (JCPDS no. 06-0395), SnO_2_ (tetragonal, JCPDS no. 41-1445), Ag (JCPDS no. 004-0783), Ag_3_Sn (JCPDS no. 071-0530), and Ag_4_Sn (JCPDS no. 029-1151) are shown in black. The black box is for visual guidance to the peaks in the samples which were assigned as intermetallic compounds labelled with filled circles.

Fig. 1 shows the XRD data for the as-synthesized β-Sn NPs and Sn/Ag–Sn NPs using various molar ratios of Sn and Ag. The synthesized Sn NPs had peaks in 2*θ* equal to 30.645°, 32.019°, 43.872°, 44.903°, 55.332°, 62.540°, 63.785°, and 64.578°; all peaks can be indexed to the peaks of β-Sn. For Sn/Ag–Sn NPs, three additional small peaks were observed in the area marked with the rectangular box. The peaks at 2*θ* equal to 34.657°, 37.603°, and 39.589° correspond to peaks of the Ag_3_Sn intermetallic compound. However, these three peaks may be also indexed to peaks of the Ag_4_Sn intermetallic compound. Ag_4_Sn intermetallic compound has peaks in 2*θ* equal to 34.911°, 37.588°, and 39.801°, which resemble those of Ag_3_Sn. No metallic Ag peak was observed. The XRD pattern of the coated NPs confirmed the presence of the Ag–Sn intermetallic compound (Ag_3_Sn and/or Ag_4_Sn) in the NPs. When a higher ratio of the input Ag cations was used, more intense XRD peaks of the intermetallic compounds were observed. However, the peaks of Sn were more significant than those of the intermetallic compounds, suggesting that the Sn/Ag–Sn NPs were mainly dominated by Sn with smaller amounts of Ag–Sn intermetallic compounds. It should be noted that the solvent has no reducing power to Ag cations. Thus, the existence of the intermetallic compounds suggests that the galvanic reaction occurred and Ag cations were reduced by Sn. The formation of the Ag–Sn intermetallic compounds as observed at room temperature *via* the galvanic reaction of Sn NPs and Ag cations in an organic solvent is the key result. The use of Ag for coating Sn nanoparticles has always been reported at elevated temperature without any reducing agent or at room temperature in the presence of reducing agent or in solvents which possess reducing power, *i.e.*, hexylamine, oleylamine and ethanol.^19,21,26–29^ The formation of the intermetallic compound between Ag and Sn has been achieved at room temperature without any reducing agent but it was carried out through vacuum evaporation of Sn on Ag thin film.^23,24^ Our observation demonstrated that intermetallic compounds were formed in the organic solvent under the galvanic reduction of the Ag cation by Sn at room temperature. The formation and distribution of the intermetallic compounds on Sn NPs will be analysed and discussed more in depth in the following sections.

**Fig. 2:**
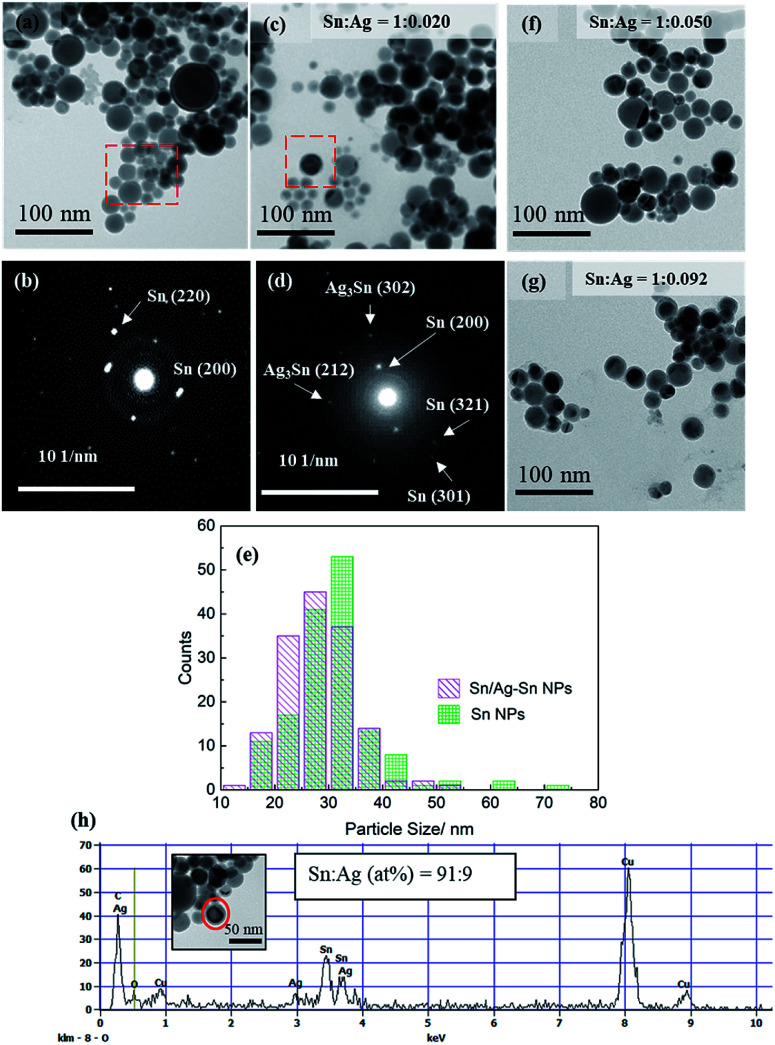
(a) TEM image and (b) SAED of Sn NPs. (c) TEM image and (d) SAED pattern of Sn/Ag–Sn NPs (Sn : Ag = 1 : 0.020 (mol/mol)). (e) Size distributions of Sn NPs and Sn/Ag–Sn NPs shown in (a) and (c), respectively. (f) TEM image of Sn/Ag–Sn NP (Sn : Ag = 1 : 0.050 (mol/mol)), (g) TEM image of Sn/Ag–Sn NP (Sn : Ag = 1 : 0.092 (mol/mol)) and (h) EDX spectra of an Sn/Ag–Sn NP (Sn : Ag = 1 : 0.020 (mol/mol)). The Cu signals in EDX spectrum came from the collodion-coated Cu grid used for preparing the sample.

Fig. 2 shows the TEM images of the as-synthesized spherical Sn NPs (Fig. 2a) and the Sn/Ag–Sn NPs (Fig. 2c) with Sn : Ag = 1 : 0.020 (mol/mol). The Sn NPs used for coating had an average size of 31.4 ± 8.2 nm (Fig. 2e). The bright spots in the SAED pattern of Sn NPs (Fig. 2b) were indexed to planes of β-Sn, confirming that the Sn NPs were β-Sn. There was a thin layer around the NPs which could be amorphous tin oxides since no crystalline tin oxides were observed in either XRD or SAED patterns. The oxide layers might be formed during purification when the Sn NPs were exposed to air during solvent exchange, as reported for Sn NPs synthesized using the same method by Kravchyk and coworkers.^5^ The Sn/Ag–Sn NPs remained spherical after galvanic reaction for all the input molar ratios of Sn and Ag (Fig. 2c, f, and g). The average size of the NPs was 28.4 ± 6.3 nm (Fig. 2e) for Sn : Ag = 1 : 0.020 (mol/mol), similar to Sn NPs which were used as the core. The bright spots in the SAED pattern of the NPs (Fig. 2d) could be indexed to planes of β-Sn and Ag–Sn intermetallic compounds (Ag_3_Sn and/or Ag_4_Sn). EDX point analysis for a single Sn/Ag–Sn NP (Fig. 2h) further proved that elemental Ag was present in the coated Sn NP. There was no yolk–shell structure observed in TEM images suggesting that the galvanic reaction occurred with the inward diffusion of the Ag cation into the Sn core and outward diffusion of the Sn cation through the oxide shell at room temperature. Kriegner *et al.* have reported similar observations when the reaction was performed in oleylamine at a higher temperature.^22^

### 3.2. HR-TEM, HAADF, and elemental mapping of Sn/Ag–Sn NPs

**Fig. 3:**
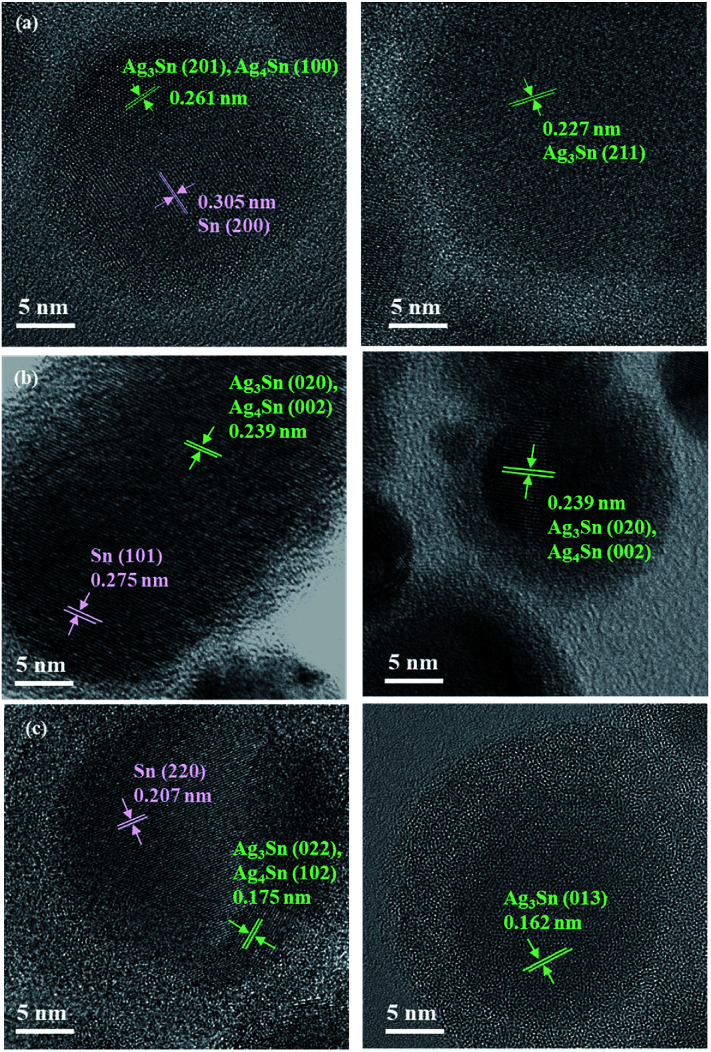
HR-TEM images of Sn/Ag–Sn NPs with various input molar ratios of Sn and Ag, (a) Sn : Ag = 1 : 0.020, (b) 1 : 0.050, and (c) 1 : 0.092 (mol/mol), showing both Janus and uniform structures. Green lines and arrows are for the Ag–Sn portion in the NPs while pink lines and arrows indicate the Sn portion.

To understand the fine structure of the Sn/Ag–Sn NPs, HR-TEM was used. Fig. 3 shows the HR-TEM images of Sn/Ag–Sn NPs with the input ratio of Sn : Ag = 1 : 0.020, 1 : 0.050, and 1 : 0.092 (mol/mol). Based on both TEM and HR-TEM images, the Sn/Ag–Sn NPs were categorized into either a uniform structure or a Janus structure. For uniform structures, most NPs showed lattice spacings, which were assigned to Ag–Sn (Fig. 3, Tables S1–S3†). The lattice of such NPs was indexed to the planes of Ag_3_Sn or Ag_4_Sn. In this case, the Ag^+^ ions, which were injected into the solution, reacted completely with the surface of Sn NPs to form the intermetallic compound. In contrast, there were some NPs exhibiting the uniform structure but their lattice spacings were indexed to the (200) and (220) planes of pure β-Sn (Fig. S2†). In this case, the Sn NPs did not react with Ag^+^ ions to form the Ag–Sn intermetallic compound. This might be due to insufficient Ag^+^ in the reaction solution.

**Fig. 4:**
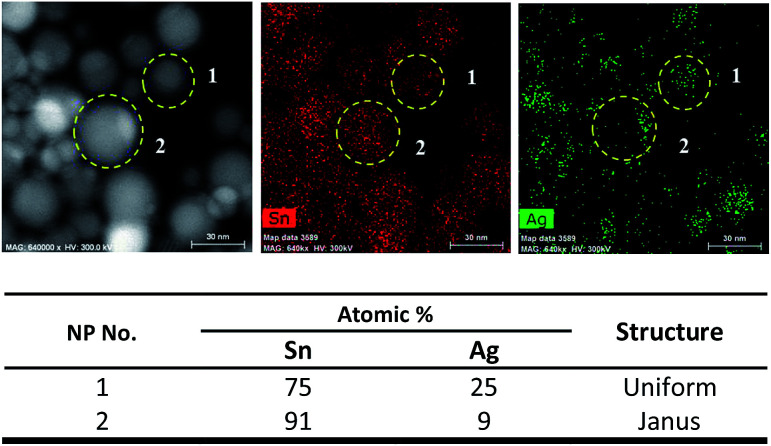
HAADF, elemental mapping and table corresponding to the atomic percentages of Sn and Ag in Sn/Ag–Sn NPs number 1–2 (information on NPs number 3–15 is available in Fig. S3 in ESI†).

For Sn/Ag–Sn NPs exhibiting a Janus structure, the lattice of the NPs was indexed to (200) plane of β-Sn, (201) plane of Ag_3_Sn or (100) plane of Ag_4_Sn. In Janus structures, a portion of the NPs was the Ag–Sn intermetallic compound, while the other portion was pure β-Sn. The existence of a Janus structure also suggests that its formation could be attributed to a deficiency of Ag^+^ ions available to react with Sn NPs. Since the coated NPs might be full Ag–Sn intermetallic compound NPs, HAADF and elemental mapping analysis were performed to understand the composition of Sn and Ag in the coated NPs. Fig. 4 shows the HAADF and corresponding elemental mapping of both uniform and Janus NPs which exhibited the intermetallic compound.

A NP will consist of 24–25 at% (atomic percentages) Sn and 75–76 at% Ag if it is an ε-Ag_3_Sn NP; and 12–22 at% Sn with 78–88 at% Ag if it is a ζ-Ag_4_Sn NP. Based on elemental mapping data of several uniform NPs (Fig. 4 and Fig. S3†), the atomic percentage of Sn was always higher than Ag, confirming that uniform NPs were dominated by the Sn element. This result suggested that the NPs had a Sn core with an Ag–Sn intermetallic compound shell instead of being fully Ag–Sn intermetallic compounds. There were several uniform NPs (particle numbers 6, 13, and 14 in Fig. S3†) which had higher atomic percentage of Ag than that of Sn. However, this was less than the 75–76 at% Ag of the Ag–Sn intermetallic compound. These types of NPs might have a smaller Sn core with a thick intermetallic shell. However, this type of NPs was less frequently observed in the overall sample of NPs. For Janus NPs, all the NPs (numbers 2, 4, 7, 8, and 12 in Fig. S3†) had a higher atomic percentage of Sn. There are two portions which were assigned to β-Sn and the Ag–Sn intermetallic compound, respectively, in a Janus structure NP. Elemental mapping analysis on the Ag–Sn portions of the Janus NPs (Fig. S4†) showed that they had higher atomic percentages of Sn than that of the Ag, *i.e.*, Sn : Ag = 68 : 32 for the Janus NP number 2 in Fig. 4. This result suggests that the Ag–Sn portion of the NPs consisted of a Sn core with an Ag–Sn shell. In short, Janus structure NPs could be depicted as Sn NPs partially coated with an Ag–Sn intermetallic shell.

### 3.3. Effect of Ag^+^ cations concentrations on the structure of Sn/Ag–Sn NPs

**Fig. 5:**
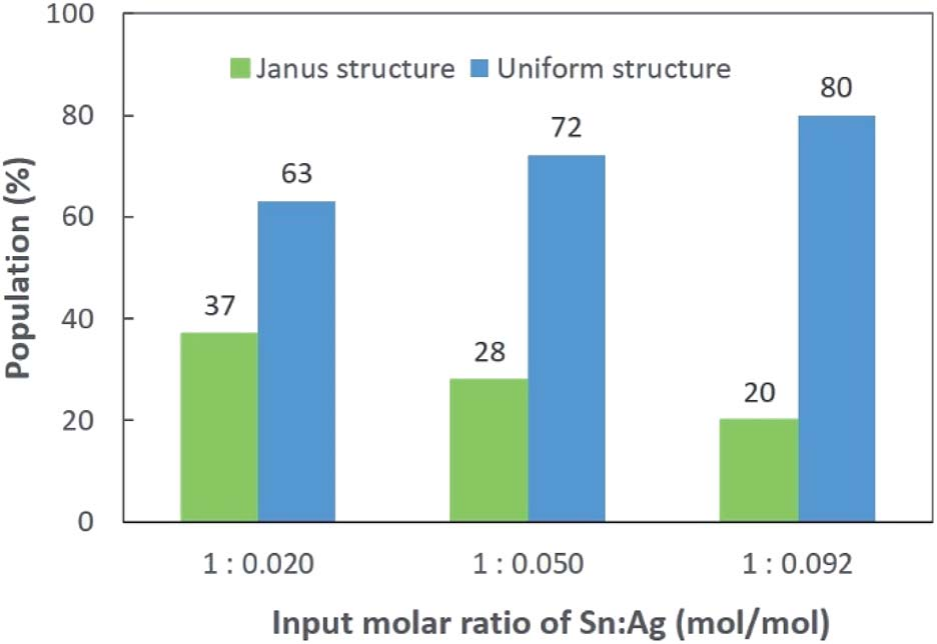
Distribution of Janus and uniform structures of Sn/Ag–Sn NPs with various input molar ratios of Sn and Ag, *i.e.*, Sn : Ag = 1 : 0.020, 1 : 0.050, and 1 : 0.092 (mol/mol).

The Sn/Ag–Sn NPs synthesized by using an input ratio of Sn : Ag = 1 : 0.020 (mol/mol) were either pure β-Sn NPs, β-Sn NPs partially coated or fully coated with an Ag–Sn shell. This result suggests that at such a low input ratio of Ag^+^ cations compared with Sn, the availability of Ag might be insufficient to allow all Sn NPs to be fully coated with an Ag–Sn shell. The effect of Ag^+^ concentrations on the distribution of uniform and Janus structure NPs was studied using TEM images by identifying the NPs that had a clear Janus structure and the results are shown in Fig. 5. When higher concentration of Ag^+^ was present in the solution, Janus structures still existed (Fig. 3b and c), but the number of NPs with the Janus structure significantly decreased. When the input ratio of Ag was increased, *i.e.*, Sn : Ag varied from 1 : 0.020 (mol/mol) to 1 : 0.092 (mol/mol), the percentage of NPs with the Janus structure decreased from 37% to 20%. This suggests that a higher Ag^+^ concentration will produce more uniform structures and fewer Janus structure NPs after coating. Since the percentage of Janus NPs decreased and the relative intensity of XRD peaks of the intermetallic compound (Fig. 1) increased with increasing Ag^+^ input ratios, we conclude that the Sn NPs were coated with an Ag–Sn shell and more uniform coating were achieved with a higher input ratio of Ag^+^.

Based on the elemental mappings of NPs (Fig. 4, S3 and S5†), the average atomic percentage of Ag in uniform NPs increased from 28% to 45% when Sn : Ag varied from 1 : 0.020 (mol/mol) to 1 : 0.092 (mol/mol). With a higher concentration of Ag^+^ present in the solution, more Ag^+^ can be reduced to Ag atoms. More Ag atoms were then available for interdiffusion between Sn and Ag to form the Ag–Sn intermetallic shell on β-Sn cores. When the concentration of Ag^+^ increased, some NPs were transformed to full Ag–Sn NPs, although most of the NPs still possessed a Sn core with an Ag–Sn shell structure. Since the amount of Ag was still deficient to fully cover the surfaces of all NPs, some NPs remained partially coated with the Ag–Sn shell (Janus structure) while some were pure Sn NPs.

### 3.4. Formation of Ag–Sn intermetallic shell on Sn NPs

**Fig. 6:**
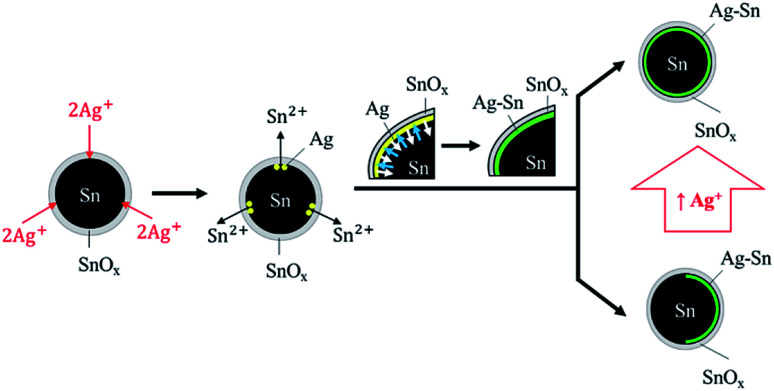
Coating of Ag–Sn intermetallic compound on Sn NPs *via* sequential steps: reduction of Ag^+^ by Sn and interdiffusion of Sn and Ag (Sn into Ag as shown by blue arrows; Ag into Sn as shown by white arrows). More uniform Sn/Ag–Sn NPs can be obtained with higher concentration of Ag^+^.

From all the syntheses, it was evident that the Ag–Sn intermetallic compound formed on Sn NPs regardless of the concentration of Ag^+^. The formation of the Ag–Sn intermetallic compound can be explained by the operation of two sequential processes (Fig. 6).

First, Ag^+^ ions that penetrated through the surface tin oxide were reduced to Ag atoms by the Sn atoms on the surface of Sn NPs *via* galvanic reaction. Sn^2+^ ions were released during this reaction through the tin oxide shell. Once Ag atoms formed, the Sn atoms on the surface of NPs then diffused into Ag to form the Ag–Sn intermetallic compound. This is because the diffusion rate of low-melting point metals such as Sn into Ag is high; the interdiffusion coefficient is approximately 3.4 × 10^−16^ cm^2^ s^−1^.^23,24^ The large difference in surface energy of Sn (0.675 J m^−2^) and Ag (1.250 J m^−2^) is the reason for the upward diffusion of Sn into the growing Ag layer.^30–32^ The formation of the intermetallic compound will be completed faster with the presence of higher concentrations of Sn, which was the typical condition of our experiments wherein the maximum molar input of Ag was less than 1% of Sn. Clearly, Ag–Sn intermetallic compounds were easily formed, even at room temperature.

**Fig. 7:**
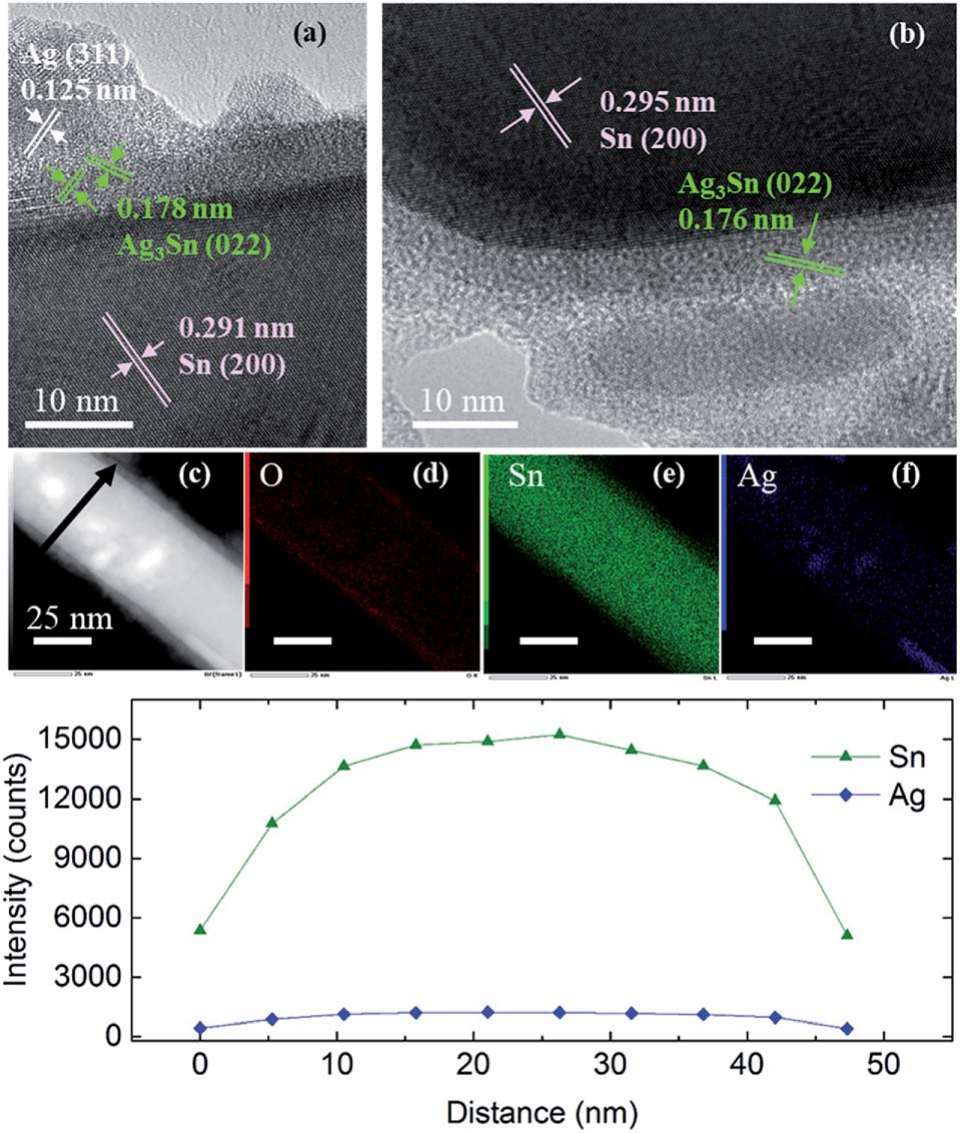
(a and b) HR-TEM images of Sn/Ag–Sn NRs, (c) HAADF, (d–f) elemental mappings of the NR shown in (c), and (g) line profiles along the arrow in (c).

Similar observations were obtained when using Sn NRs as the Sn core for coating (Fig. S6–S8†). The Sn NRs before coating (Fig. S7†) had a tin oxide layer on the surface and some small spherical Sn NPs existed along with NRs in the sample. Fig. 7 shows the HR-TEM images of Sn/Ag–Sn NRs. Based on these images, the lattices of coated NRs were indexed to (200) of β-Sn, (022) of Ag_3_Sn and (311) of Ag (Table S4†). The inner part of the NRs was made up of Sn. There was a thin layer of intermetallic compound on the Sn core. On the surface of the intermetallic compound layer, Ag islands were observed. Elemental mapping and line scan on the coated NRs (Fig. 7) revealed that elemental Ag was present on all the surface area of NRs. Since Ag had the same location as the Sn, it was reasonable to propose that the NRs were covered by Ag–Sn intermetallic compound.

The results observed in Sn/Ag–Sn NRs also suggested that the Ag^+^ ions, which were injected into the solution, reacted completely with Sn on the surface of NRs to form an intermetallic compound layer. As observed in the coating using spherical Sn NPs as the core, some NRs were not coated. The important finding here is that even at room temperature, intermetallic compounds were formed on Sn NRs, below an amorphous layer, which may have been composed of tin oxides. The presence of Ag NPs was observed on the uppermost surface of Sn coated with Ag–Sn and an amorphous layer. This Ag is thought to be the product of the complete reduction of small spherical Sn NPs and Ag^+^ in the reaction solution due to the higher surface area of small spherical Sn NPs and the higher amount of Ag^+^ used, along with a long reaction time. In addition, the binding energy of Ag–Ag is around 163 kJ mol^−1^, which is higher than that of the Ag–Sn bond, which is approximately 136 kJ mol^−1^.^33^ This is possibly the reason for Ag atoms to bond with each other to form Ag NPs. In a recent study of coating Ag onto Sn NRs, the as-synthesized Sn/Ag NRs consisted of only Sn NRs with Ag NPs dispersed on the surface of NRs and no intermetallic compound existed.^34^ These observations are similar with our current study, however, the presence of a very thin layer of intermetallic compound between Sn NRs and Ag NPs was likely to have been overlooked in their study.

## Conclusions

Coating of a layer of Ag–Sn intermetallic compounds on Sn NPs was achieved at room temperature *via* galvanic reaction. The Sn/Ag–Sn NPs retained the original shape of Sn and had a similar particle size after coating. The coated spherical Sn NPs were either uniform structure NPs or Janus structure NPs, wherein the uniform structure was comprised mainly of Sn/Ag–Sn core/shell NPs with some pure Sn NPs and the Janus structure composed of a Sn core with a partially coated Ag–Sn intermetallic shell. The Ag–Sn intermetallic shell was formed *via* two sequential steps. First, Ag^+^ ions penetrated through the surface oxide layer of Sn NPs and were reduced to Ag by Sn *via* galvanic reaction. Then, interdiffusion between Sn and Ag occurred to form an Ag–Sn intermetallic shell. Due to the presence of insufficient Ag^+^ ions in the solution, some Sn NPs were partially coated while some were not coated. By increasing the concentration of Ag^+^ in the solution, more uniform Sn/Ag–Sn NPs could be obtained.

## Conflicts of interest

There are no conflicts to declare.

## Supplementary Material

RA-009-C9RA02987G-s001
