# Supplementary material for: Synthesis of Sn/Ag–Sn nanoparticles via room temperature galvanic reaction and diffusion
Source: RSC Adv. 2019 Jul 12;9(38):21786–92. doi: 10.1039/c9ra02987g (PMC9066527; doi:10.1039/c9ra02987g)
Supplement: RA-009-C9RA02987G-s001 [file RA-009-C9RA02987G-s001.pdf]

Electronic Supplementary Material (ESI) for RSC Advances.
This journal is © The Royal Society of Chemistry 2019

Supporting Information

Synthesis of Sn/Ag-Sn nanoparticles via room temperature galvanic reaction and diffusion

Min Jia Saw, Mai Thanh Nguyen, Shilei Zhu, Yongming Wang, and Tetsu Yonezawa*

Division of Materials Science and Engineering, Faculty of Engineering, Hokkaido University, Kita 13 Nishi 8, Kita-ku, Sapporo, 060-8628, Japan

Email: testu@eng.hokudai.ac.jp

Experimental Section

Preparation of Sn NPs as core

Spherical Sn NPs as the core were synthesized based on the modified method with the reference to Kravchyk et al.[1] For the preparation of spherical Sn NPs, oleylamine as solvent was added into two neck Kjeldahl-shaped flask and heated up to 140 °C for 1 h under vacuum. The solution was then cooled down to 50 °C. Tin(II) chloride was then added into the solution and stirred until dissolve. The stirring speed was kept at 800 rpm throughout the synthesis. The solution was then heated up to 140 °C for another 30 min under vacuum. The solution was further heated up to 180 °C under argon, followed by the injection of lithium bis(trimethylsilyl)amide into the solution to form Sn-oleylamide species. Within 10 s, DIBAH was then injected into the solution to reduce the species into Sn. The solution was subsequently left to react for 1 h at 180 °C under argon to form Sn NPs. After the reaction, the solution was quenched down to room temperature using ice-water bath. When the temperature dropped to around 130 °C, anhydrous toluene was added into the solution to stop the reaction. The reacted solution was then centrifuged using 1-propanol at 8000 rpm for 4 min. The purification process was repeated twice to obtain pure Sn NPs.

Preparation of Sn nanorods (NRs) as core

Sn NRs as core were synthesized based on the method proposed by Juan et al.[2] 1-Propanol (99.5% purity, Junsei Chemical) was dehydrated using molecular sieves (3A 1/16) before use. First, 3.0728 g PVP K-90 (MW 630000 with 95% purity, Tokyo Chemical Industry) was added into 50 mL 1-propanol in a two-necked Kjeldahl-shaped flask and stirred for 15 min. The stirring speed was kept at 800 rpm throughout the synthesis. Tin(II) acetate (97% purity, Wako Chemical) of 0.36 g was then added into the solution and stirred for 1 h. At the same time, 0.88625 g sodium borohydride (95% purity, Wako Chemical) was added into 15 mL 1-propanol in another three-necked round bottom flask and stirred until dissolved. Solution containing Sn precursor and PVP was cooled down to -15 ℃ and sodium borohydride solution was cooled down to 0 ℃. Then, 10 mL cooled sodium borohydride solution was injected into the solution containing tin precursor. The solution was subsequently left to react for 44 h at -15 ℃. The reacted solution was then centrifuged using 1-propanol at 15000 rpm for 30 min. The purification process was repeated three times to obtain pure Sn NRs.

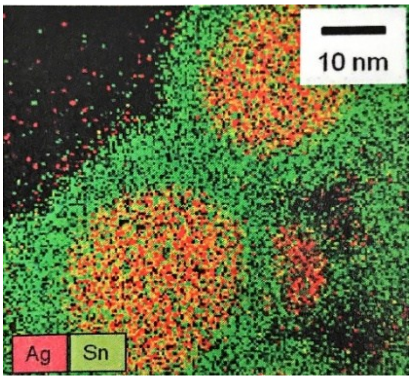

Figure S1. Elemental mapping of the resulting Sn nanoparticles with silver nitrate at high temperature. The core consisted of Ag-Sn intermetallic compound and the shell consisted of Sn. Reproduced with permission from reference 3, copyright Hokkaido University, 2016.

Table S1: Calculated lattice parameter of Sn/Ag-Sn NPs (Sn:Ag = 1:0.020 (mol/mol)) based on HR-TEM images in Figure 3(a) of main text.

Structure	d-calculated [nm]	d-reference [nm]	(*hkl*)
Janus	0.305	0.291	Sn (*200*)
	0.261	0.258<br>0.257	Ag_3Sn (*201*)<br>Ag_4Sn (*100*)
Uniform	0.229	0.227	Ag_3Sn (*211*)

Table S2: Calculated lattice parameter of Sn/Ag-Sn NPs (Sn:Ag = 1:0.050 (mol/mol)) based on HR-TEM images in Figure 3(b) of main text.

Structure	d-calculated [nm]	d-reference [nm]	(*hkl*)
Janus	0.275	0.279	Sn (*101*)
	0.239	0.239<br>0.239	Ag_3Sn (*020*)<br>Ag_4Sn (*002*)
Uniform	0.239	0.239<br>0.239	Ag_3Sn (*020*)<br>Ag_4Sn (*002*)

Table S3: Calculated lattice parameter of Sn/Ag-Sn NPs (Sn:Ag = 1:0.092 (mol/mol)) based on HR-TEM images in Figure 3(c) of main text.

Structure	d-calculated [nm]	d-reference [nm]	(*hkl*)
Janus	0.207	0.206	Sn (*220*)
	0.175	0.176<br>0.175	Ag_3Sn (*022*)<br>Ag_4Sn (*102*)
Uniform	0.162	0.163	Ag_3Sn (*013*)

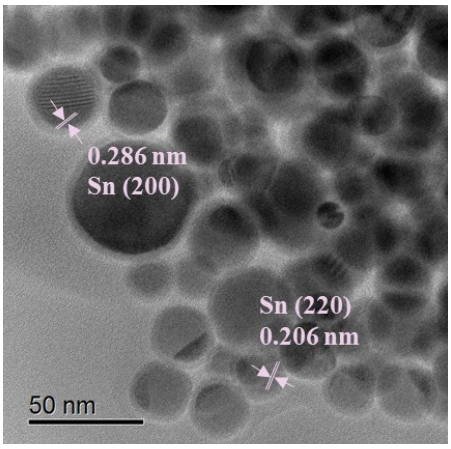

Figure S2. HR-TEM image of uniform nanoparticles in Sn/Ag-Sn NP sample (Sn:Ag = 1:0.020 (mol/mol). The lattice spacings of the NPs can be index to (200) and (220) of pure β-Sn, indicating that these NPs did not react with Ag^+ ions to form Ag-Sn intermetallic compound.

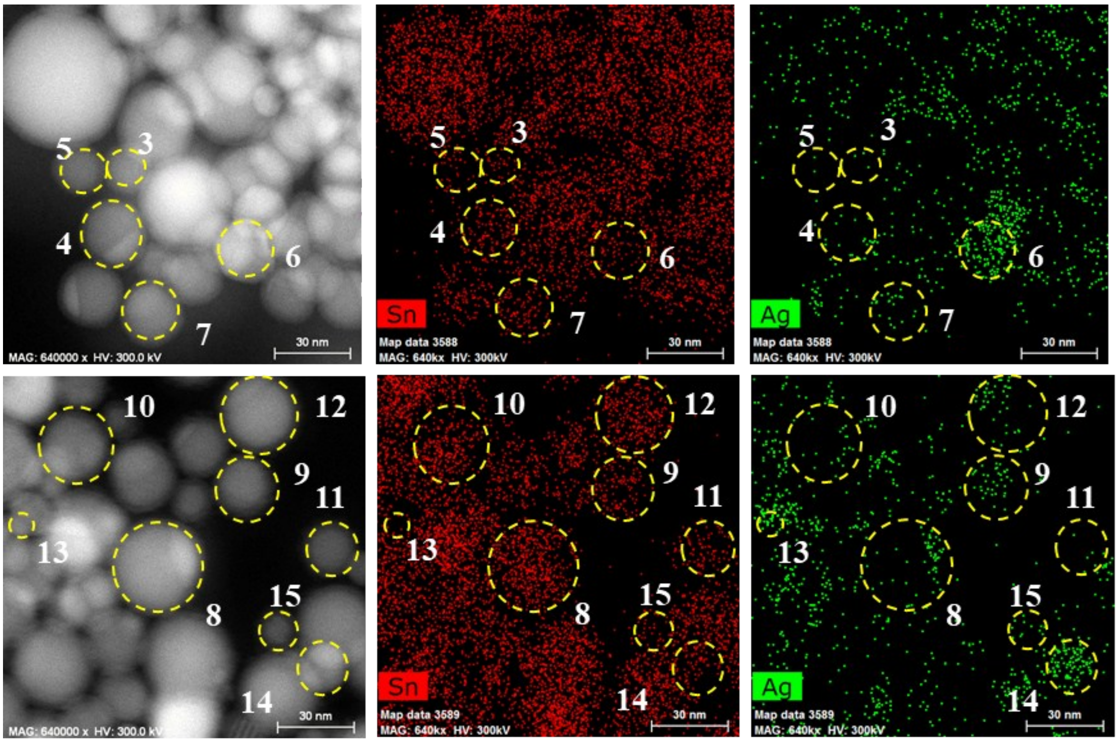

NP No.	Atomic %		Structure
	Sn	Ag	
**3**	99	1	Uniform
**4**	83	17	Janus
**5**	89	11	Uniform
**6**	39	61	Uniform
**7**	81	19	Janus
**8**	91	9	Janus
**9**	75	25	Uniform
**10**	90	10	Uniform
**11**	92	8	Uniform
**12**	86	14	Janus
**13**	38	62	Uniform
**14**	44	56	Uniform
**15**	79	21	Uniform

Figure S3. HAADF, elemental mapping images and table corresponding to atomic percentage of Sn and Ag in Sn/Ag-Sn NPs (Sn:Ag = 1:0.020 (mol/mol)).

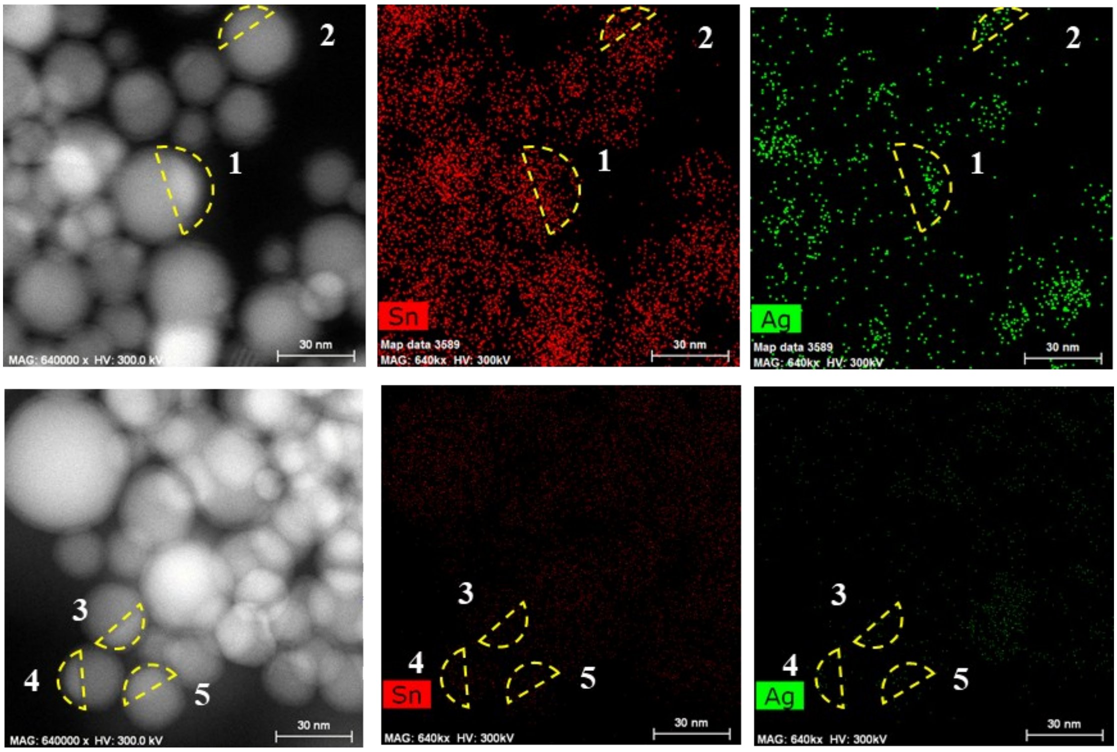

NP No.	Atomic % (Ag-Sn portion)	
	Sn	Ag
**1**	68	32
**2**	63	37
**3**	60	40
**4**	55	45
**5**	62	38

Figure S4. HAADF, elemental mapping images and table corresponding to atomic percentage of Sn and Ag in the Ag-Sn portion of Sn/Ag-Sn NPs (Sn:Ag = 1:0.020 (mol/mol)).

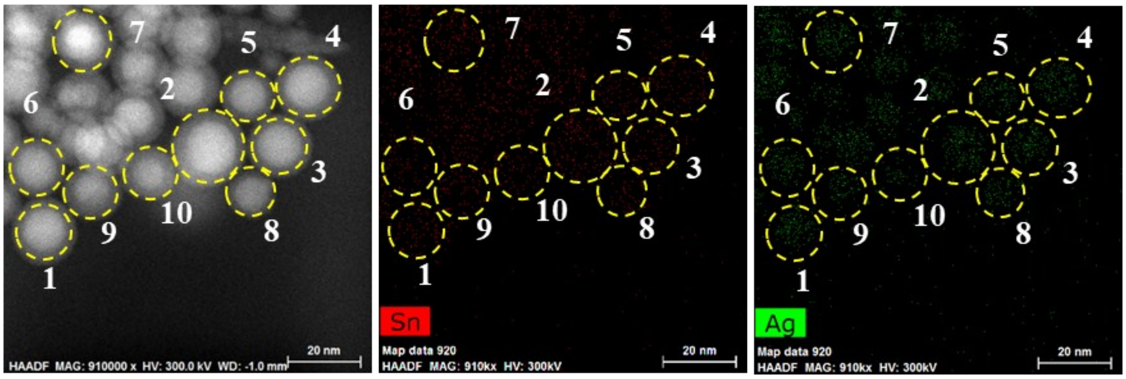

NP No.	Atomic %		Structure
	Sn	Ag	
**1**	50	50	Uniform
**2**	57	43	Uniform
**3**	53	47	Uniform
**4**	50	50	Uniform
**5**	50	50	Uniform
**6**	46	54	Uniform
**7**	59	41	Uniform
**8**	51	49	Uniform
**9**	57	43	Uniform
**10**	75	25	Uniform

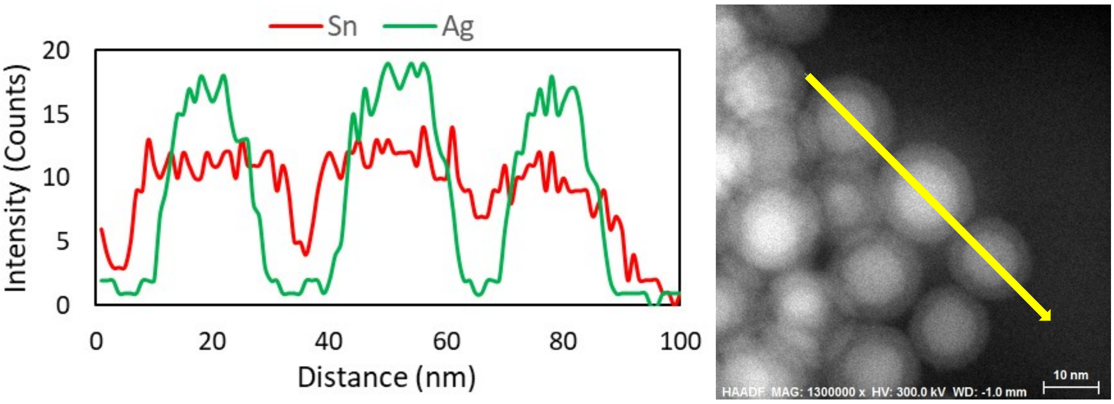

Figure S5. (Top) HAADF and elemental mapping images, (middle) table corresponding to atomic percentage of Sn and Ag of Sn/Ag-Sn nanoparticles (Sn:Ag = 1:0.092 (mol/mol)) shown in the mapping images, (bottom) EDX line-profile along the arrow shown in the HAADF image of Sn/Ag-Sn nanoparticles.

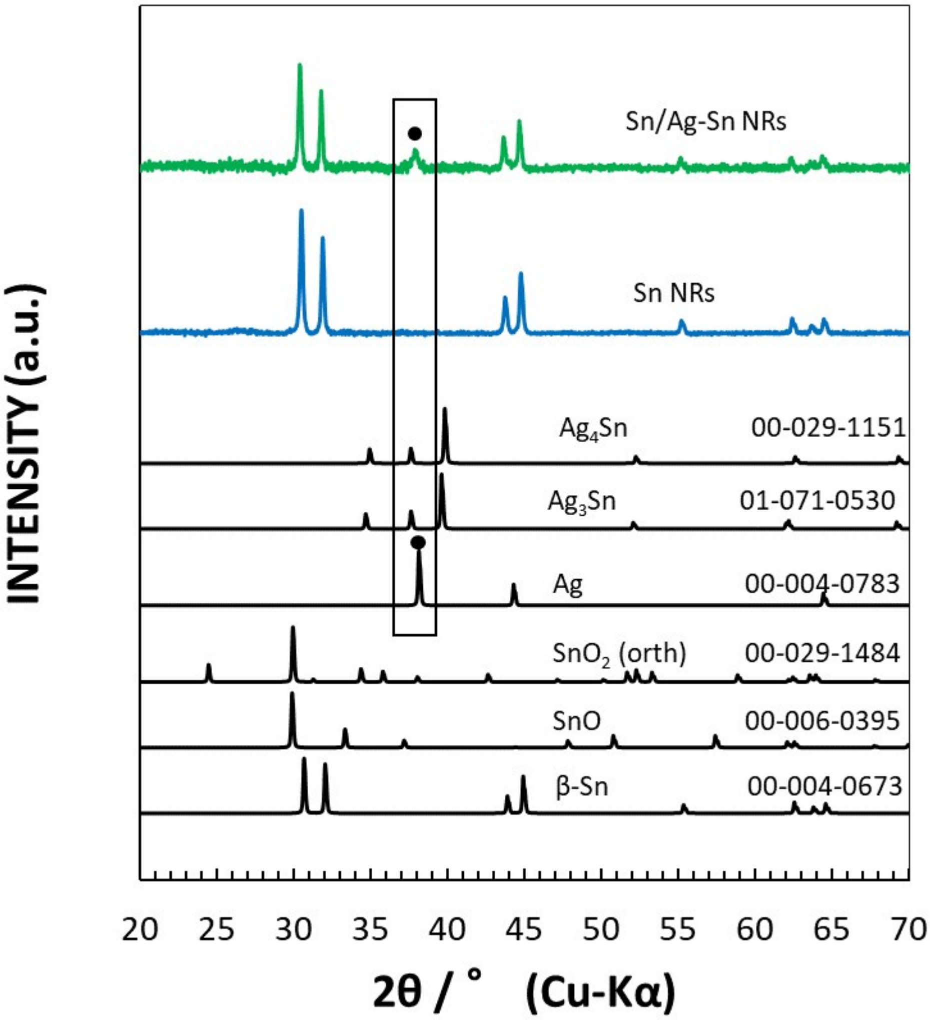

Figure S6. XRD pattern of as-synthesized Sn NRs (blue curve) and Sn/Ag-Sn NRs (green curve). Reference patterns of β-Sn (JCPDS no. 04-0673), SnO (JCPDS no. 06-0395), SnO_2 (Orthorhombic, JCPDS no. 29-1484), Ag (JCPDS no. 004-0783), Ag_3Sn (JCPDS no. 071-0530) and Ag_4Sn (JCPDS no. 029-1151) are shown in black. Black box is for visual guide of the peak in the sample which was assigned for Ag (100) labeled with filled circles.

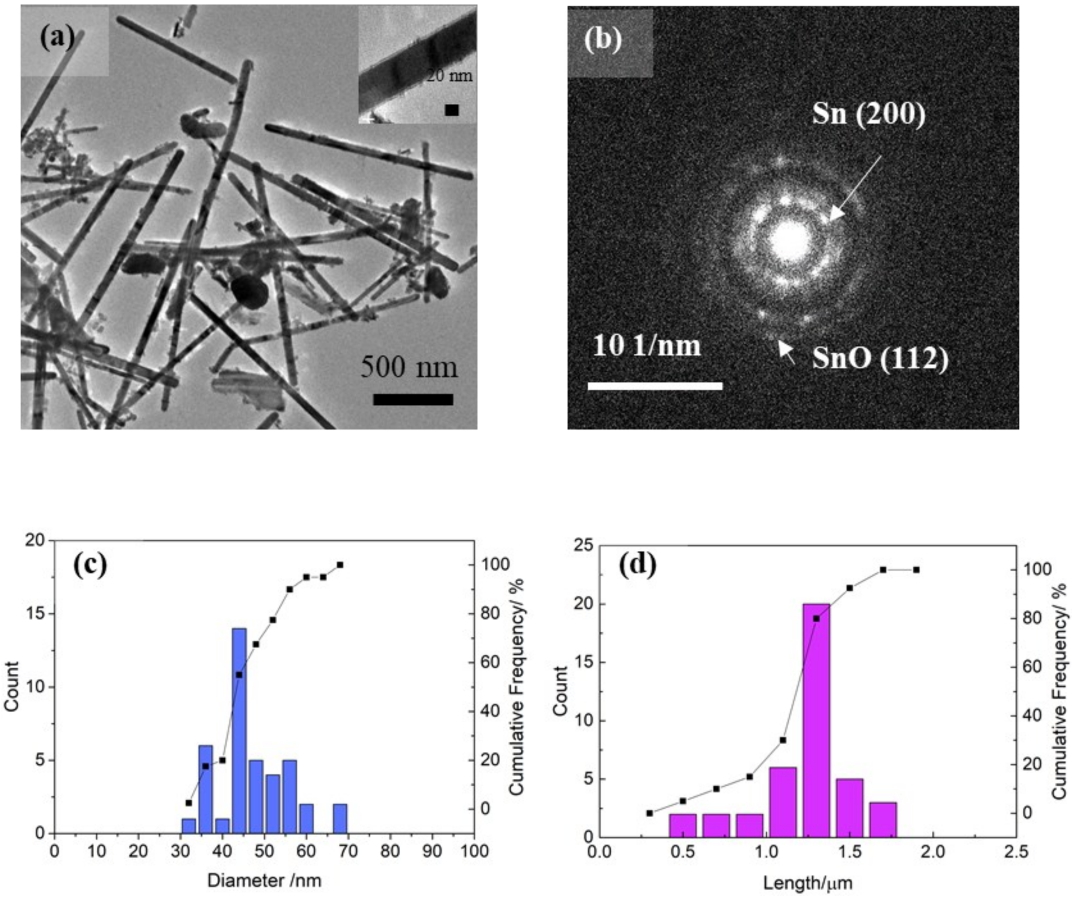

Figure S7. (a) TEM image, (b) SAED, (c) diameter and (d) length distribution of Sn NRs.

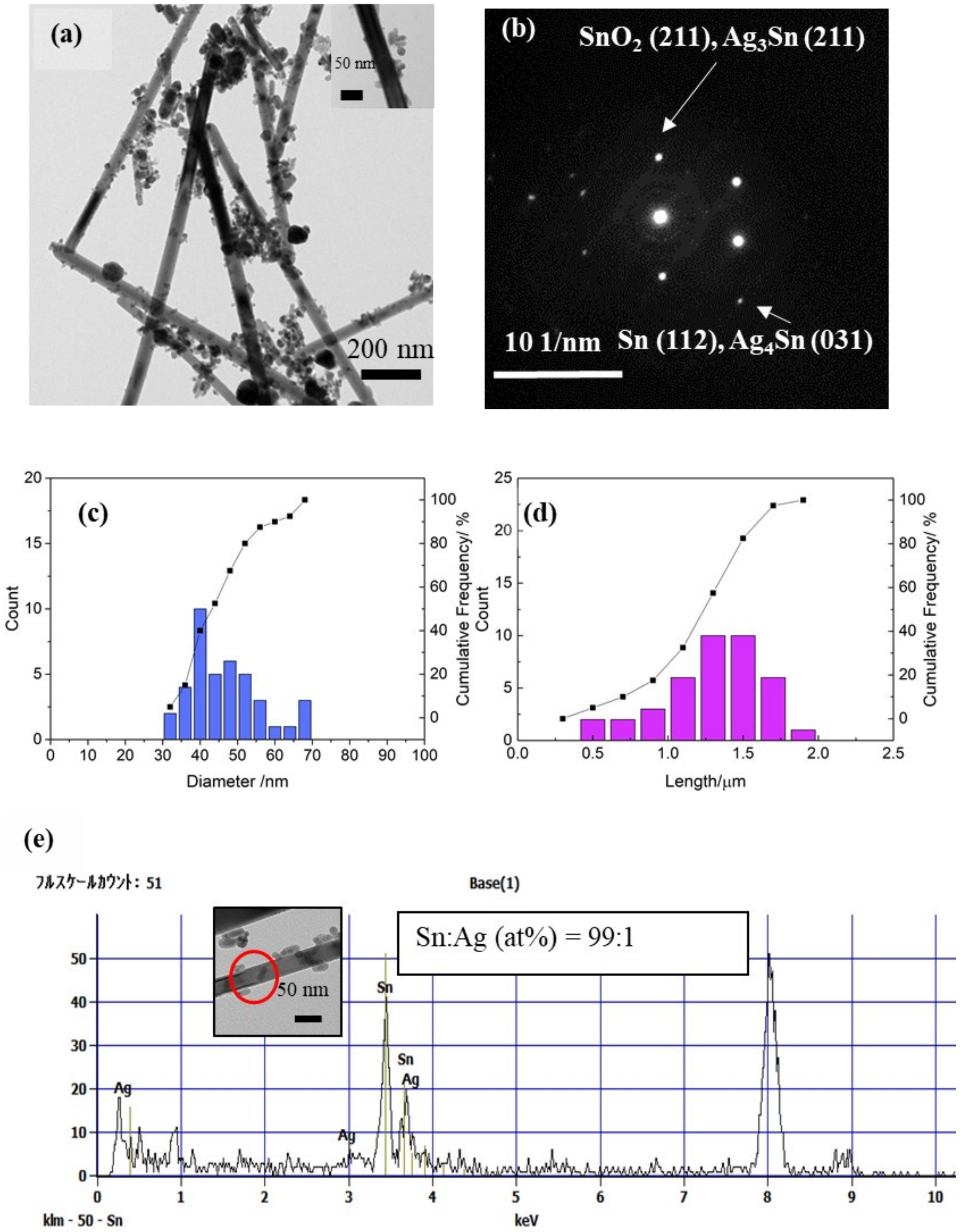

Figure S8. (a) TEM image, (b) SAED pattern, (c) diameter and (d) length distribution, and (e) EDX spectrum of red-circled Sn/Ag-Sn NRs.

Table S4: Calculated lattice parameter of Sn/Ag-Sn NRs based on HR-TEM images in Figure 7 of main text.

**Figure 7**	**d-calculated [nm]**	**d-reference [nm]**	**(*hkl*)**
(a)	0.291	0.291	Sn (*200*)
	0.178	0.176	Ag_3Sn (*022*)
	0.125	0.123	Ag (*311*)
(b)	0.295	0.291	Sn (*200*)
	0.176	0.176	Ag_3Sn (*022*)

References

[1] K. Kraychyk, L. Protesescu, M. I. Bodnarchuk, F. Krumeich, M. Yarema, M. Walter, C. Guntlin, M. V. Kovalenko, “Monodisperse and Inorganically Capped Sn and Sn/SnO_2 Nanocrystals for High-Performance Li-Ion Battery Anodes”, *J. Am. Chem. Soc.* **2013**, 135, 4199−4202.

[2] L. M. Juan, M. T. Nguyen, T. Yonezawa, T. Tokunaga, H. Tsukamoto, Y. Ishida, “Structural Control Parameters for Formation of Single-Crystalline β-Sn Nanorods in Organic Phase”, *Cryst. Growth. Des.* **2017**, 17, 4554-4562.

[3] H. Shirai, Master thesis (Hokkaido University) **2016**.
